# Connectopathy in Autism Spectrum Disorders: A Review of Evidence from Visual Evoked Potentials and Diffusion Magnetic Resonance Imaging

**DOI:** 10.3389/fnins.2017.00627

**Published:** 2017-11-09

**Authors:** Takao Yamasaki, Toshihiko Maekawa, Takako Fujita, Shozo Tobimatsu

**Affiliations:** ^1^Department of Clinical Neurophysiology, Neurological Institute, Graduate School of Medical Sciences, Kyushu University, Fukuoka, Japan; ^2^Department of Neurology, Minkodo Minohara Hospital, Fukuoka, Japan; ^3^Department of Pediatrics, Fukuoka University School of Medicine, Fukuoka, Japan

**Keywords:** autism spectrum disorder, connectopathy, visual perception, attention, visual evoked potentials, event-related potentials, diffusion tensor imaging, magnetic resonance imaging

## Abstract

Individuals with autism spectrum disorder (ASD) show superior performance in processing fine details; however, they often exhibit impairments of gestalt face, global motion perception, and visual attention as well as core social deficits. Increasing evidence has suggested that social deficits in ASD arise from abnormal functional and structural connectivities between and within distributed cortical networks that are recruited during social information processing. Because the human visual system is characterized by a set of parallel, hierarchical, multistage network systems, we hypothesized that the altered connectivity of visual networks contributes to social cognition impairment in ASD. In the present review, we focused on studies of altered connectivity of visual and attention networks in ASD using visual evoked potentials (VEPs), event-related potentials (ERPs), and diffusion tensor imaging (DTI). A series of VEP, ERP, and DTI studies conducted in our laboratory have demonstrated complex alterations (impairment and enhancement) of visual and attention networks in ASD. Recent data have suggested that the atypical visual perception observed in ASD is caused by altered connectivity within parallel visual pathways and attention networks, thereby contributing to the impaired social communication observed in ASD. Therefore, we conclude that the underlying pathophysiological mechanism of ASD constitutes a “connectopathy.”

## Introduction

Autism spectrum disorder (ASD) is a neurodevelopmental condition that is characterized by alterations in social communication and interaction; it co-occurs with restricted, repetitive patterns of behavior, interests, or activities (American Psychiatric Association, 2013). Recent neuroimaging studies have suggested that ASD is a disconnection syndrome that is associated with connectivity alterations between distributed brain areas recruited during social information processing, rather than local deficits in specific brain regions (Müller, 2008). Specifically, functional connectivity comprising long-range connections in the brain may be diminished in ASD; this is accompanied by greater localized connectivity (Geschwind and Levitt, 2007; Anderson, 2014). However, precise mechanisms underlying such abnormalities in ASD remain largely unknown.

Besides core social deficits, ASD exhibits peculiar sensory processing (e.g., superior simple and lower-level perception, but poor complex and higher-level processing) during visual (Simmons et al., 2009) and auditory (O'Connor, 2012) behavioral tasks (Yamasaki et al., 2017). In the visual aspect, ASD displays superb performance in fine-form perception (i.e., perception of local structures; Jolliffe and Baron-Cohen, 1997; Happé and Frith, 2006). In contrast, ASD is characterized by poor performance in several aspects of face (Golarai et al., 2006; Simmons et al., 2009) and motion (Spencer et al., 2000; Milne et al., 2002; Bertone et al., 2003) perception (Yamasaki et al., 2014). Various levels of visual attention deficits have been noted in ASD (Pruett et al., 2011; Amso et al., 2014). Overall, these visual perception abnormalities may contribute to social cognition impairment in ASD (Dakin and Frith, 2005).

**Figure 1:**
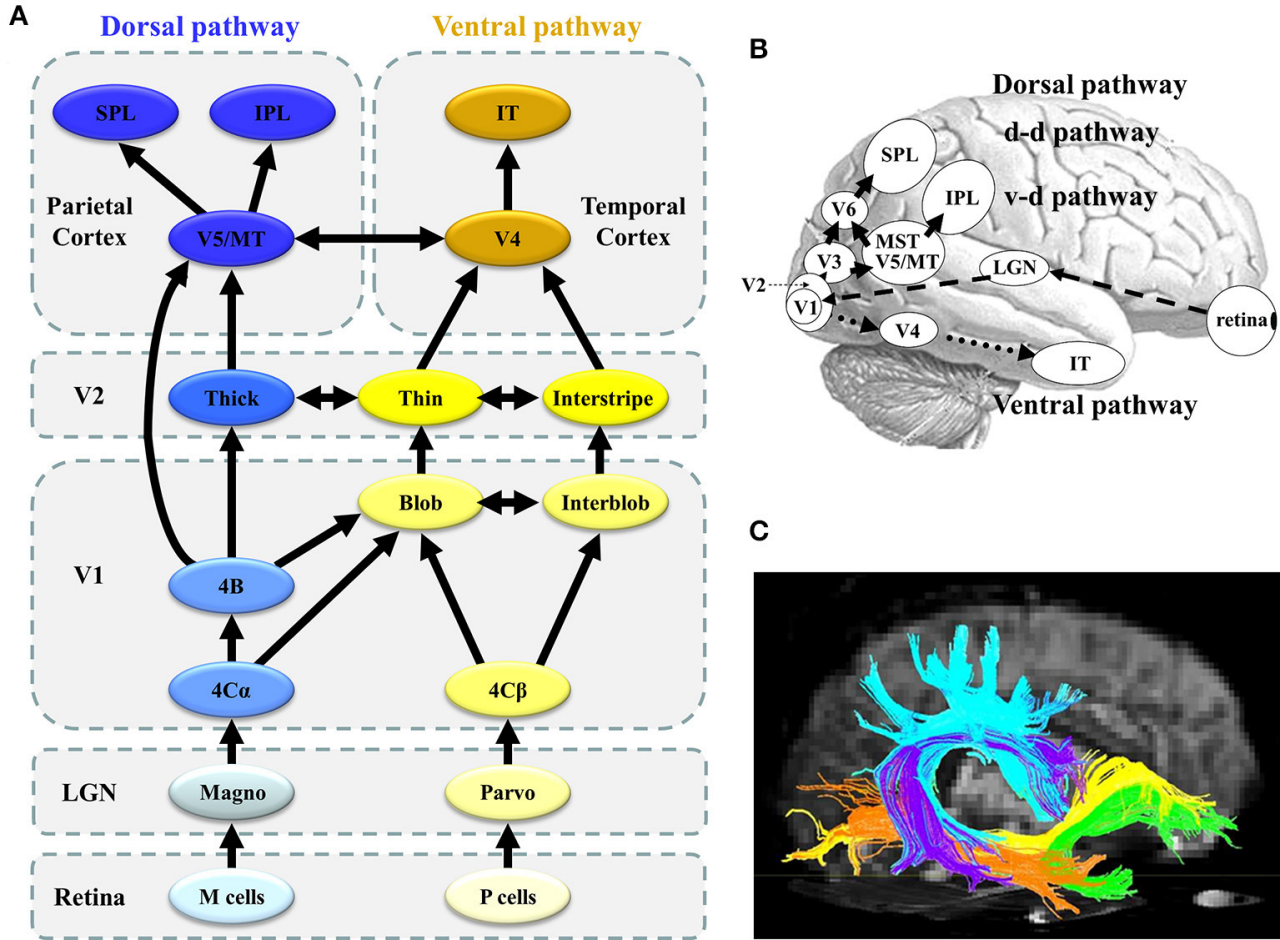
Parallel visual pathways in humans. **(A)** The monkey visual system is characterized by a set of parallel, hierarchical, multistage systems. There are two major parallel streams: parvocellular (or ventral) and magnocellular (or dorsal) pathways (see section Concept of Parallel Visual Pathways for more detailed information). **(B)** The human visual system is analogous to that of the monkey. Two major parallel streams: parvocellular (or ventral) and magnocellular (or dorsal) pathways are present. **(C)** The ventral stream corresponds to the ILF (orange) and IFOF (yellow), while the dorsal stream is related to the SLF (sky-blue) ([C] Modified from Jang, 2013 with no permission was required to reproduce). d-d pathway, dorso-dorsal pathway; v-d pathway, ventro-dorsal pathway; LGN, lateral geniculate nucleus; MT, middle temporal area; MST, medial superior temporal area; IPL, inferior parietal lobule, SPL, superior parietal lobule; IT, inferior temporal cortex; SLF, superior longitudinal fasciculus, IFOF, inferior fronto-occipital fasciculus; ILF, inferior longitudinal fasciculus.

**Figure 2:**
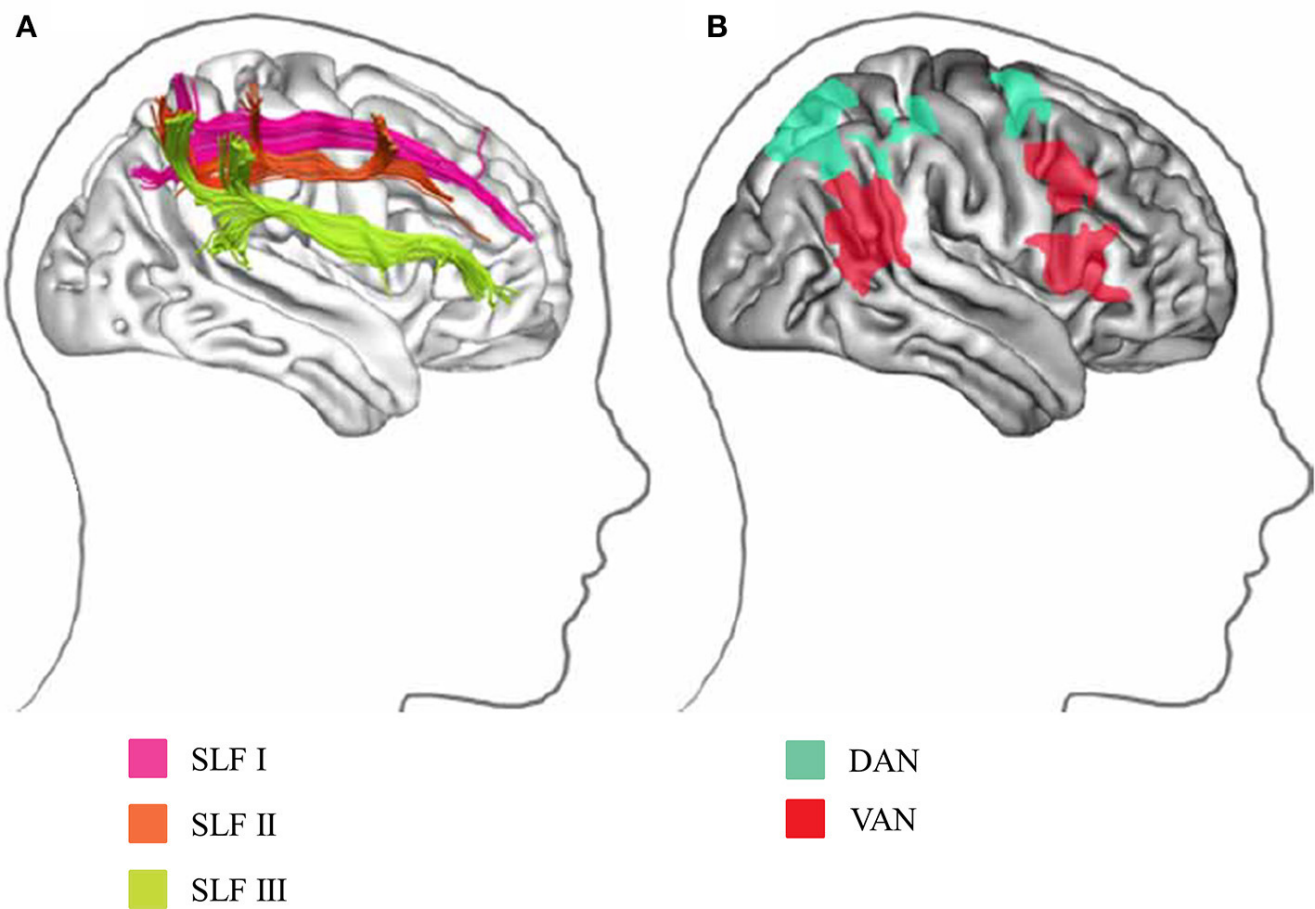
Visual attention network in humans. **(A)** Parietal and frontal areas involved in spatial attention are linked by three distinct white matter tracts in the SLF. **(B)** Projection sites in the parietal and frontal cortices correspond to the division into the DAN and VAN. DAN, dorsal attention network; VAN, ventral attention network (Modified from Bartolomeo et al., 2012 with no permission was required to reproduce).

In humans, the distinctive feature of the visual system is a set of parallel, hierarchical, multistage systems, which contribute to the processing of different visual stimulus types. Two major parallel pathways are present: parvocellular (P or ventral) and magnocellular (M or dorsal) pathways (Yamasaki et al., 2013; Figures 1A,B). These streams comprise multiple visual cortical areas and are structurally connected by fiber tracts (Figure 1C). These pathways are functionally connected by feedforward, feedback, and lateral interactions (Tobimatsu and Celesia, 2006). Moreover, the following two attention networks have been described in the human brain: ventral (bottom-up) and dorsal (top-down) frontoparietal attention networks (Corbetta et al., 2008). Both include several key nodes and are structurally connected by the periventricular white matter fiber tracts (Doricchi et al., 2008; Umarova et al., 2010; Figure 2). Therefore, it is probable that atypical visual perception in ASD is caused by alterations to the local circuitry within the visual area and connectivity between distributed visual cortical areas as well as the connectivity of attention networks.

Visual evoked potentials (VEPs) and event-related potentials (ERPs) are objective, non-invasive methods to delineate subtle functional abnormalities in the human visual system. Both VEPs and ERPs offer a direct measure of neuronal activities with high temporal resolution, in the order of milliseconds (Tobimatsu and Celesia, 2006; Yamasaki and Tobimatsu, 2014). VEP and ERP abnormalities are closely correlated with white matter damage of parallel visual pathways (Yamasaki et al., 2004). Diffusion tensor imaging (DTI) is a powerful technique that measures the white matter microstructure *in vivo*; it enables us to compute the anatomical connectivity among the brain areas (Basser and Jones, 2002). Therefore, the combined use of electrophysiological measurements (VEP and ERP) and DTI is ideally suited to examine alterations to connectivities underlying atypical visual perception in ASD.

Based on a narrative review of a series of VEP, ERP, and DTI studies conducted in our laboratory (Noriuchi et al., 2010; Fujita et al., 2011, 2013; Maekawa et al., 2011; Yamasaki et al., 2011a,b, 2013, 2014, 2017), we propose that altered connectivities (hyperconnectivity and underconnectivity) of visual and attention networks contribute to social cognition impairment in ASD. Specifically, we propose that ASD can be conceptualized as a brain network disorder that might be best characterized as a “connectopathy.”

## Concept of parallel visual pathways

As previously mentioned, both P and M pathways are important for processing the detailed visual information (Livingstone and Hubel, 1988; Nealey and Maunsell, 1994; Tobimatsu and Celesia, 2006; Yamasaki and Tobimatsu, 2012; Yamasaki et al., 2014; Figures 1A,B). Both pathways begin in the retina and there are direct anatomical connection to the primary visual cortex (V1) via the lateral geniculate nucleus. The segregation of these two pathways is perpetuated in V1. The P pathway primarily terminates in layer 4Cβ of V1 and is further differentiated into the P-blob and P-interblob pathways. The former pathway projects to the thin stripes of V2 via cytochrome oxidase (CO)-rich blobs in V1, while the latter pathway projects to the interstripes of V2 via non-CO-rich interblobs in V1. The P-blob and P-interblob pathways project to V4, and visual information is consecutively sent to the inferior temporal cortex (ventral stream). The P-blob and P-interblob streams thus act in fully different and complementary manners. The P-blob cells are involved in color discrimination. In contrast, the P-interblob cells are not color selective and respond to a line or edge of the correct orientation (form) (Livingstone and Hubel, 1988).

The M pathway primarily terminates in layer 4Cα of V1 and travels to the thick stripes of V2 via layer 4B. Cells in layer 4B most strongly respond to lines of a particular orientation. Thus, they are most selective for the direction of movement. However, cells in layer 4B lack color selectivity. The M pathway further sends their output to the V5/middle temporal area (MT) and V6 and reaches in the posterior parietal cortex (dorsal stream). The M pathway is important to detect motion and process the global structure. The dorsal stream consists of two functional streams: dorso-dorsal (d-d) and ventro-dorsal (v-d) pathways. The d-d pathway includes V6 and the superior parietal lobule (SPL), while the v-d pathway includes V5/MT and the inferior parietal lobule (IPL) (Yamasaki et al., 2012a). The distinct functions of the P-blob (color), P-interblob (form), and M pathways depend on their specific physiological properties (Livingstone and Hubel, 1988; Tobimatsu and Celesia, 2006; Table 1).

**Table 1:** Physiological characteristics of the magnocellular, parvocellular-blob, and parvocellular-interblob pathways.

**Physiology**	**Magnocellular**	**Parvocellular**	
		**Blob**	**Interblob**
Spatial frequency selectivity	Low frequency	Medium frequency	**High frequency**
Color selectivity	No	**Yes**	No
Contrast sensitivity	**High**	Low	Low
Temporal resolution	**Fast**	Slow	Slow
Conduction velocity	**Fast**	Slow	Slow

*The bold means the important characteristics*.

In relation to the white matter bundle, the ventral stream constitutes the inferior longitudinal fasciculus (ILF: orange in Figure 1C) and inferior fronto-occipital fasciculus (IFOF: yellow in Figure 1C). The former connects the occipital and temporal lobes while the latter connects the occipitoparietal and frontal regions (ffytche et al., 2010; Kravitz et al., 2013; Figure 1C). In contrast, the dorsal stream involves the inferior and superior parietal brain regions and connects with the frontal lobes via a long-range white matter bundle called the superior longitudinal fasciculus (SLF: sky-blue in Figure 1C; ffytche et al., 2010; Kravitz et al., 2011).

## VEP studies in ASD

### Parvocellular and magnocellular functions at V1 in ASD

**Figure 3:**
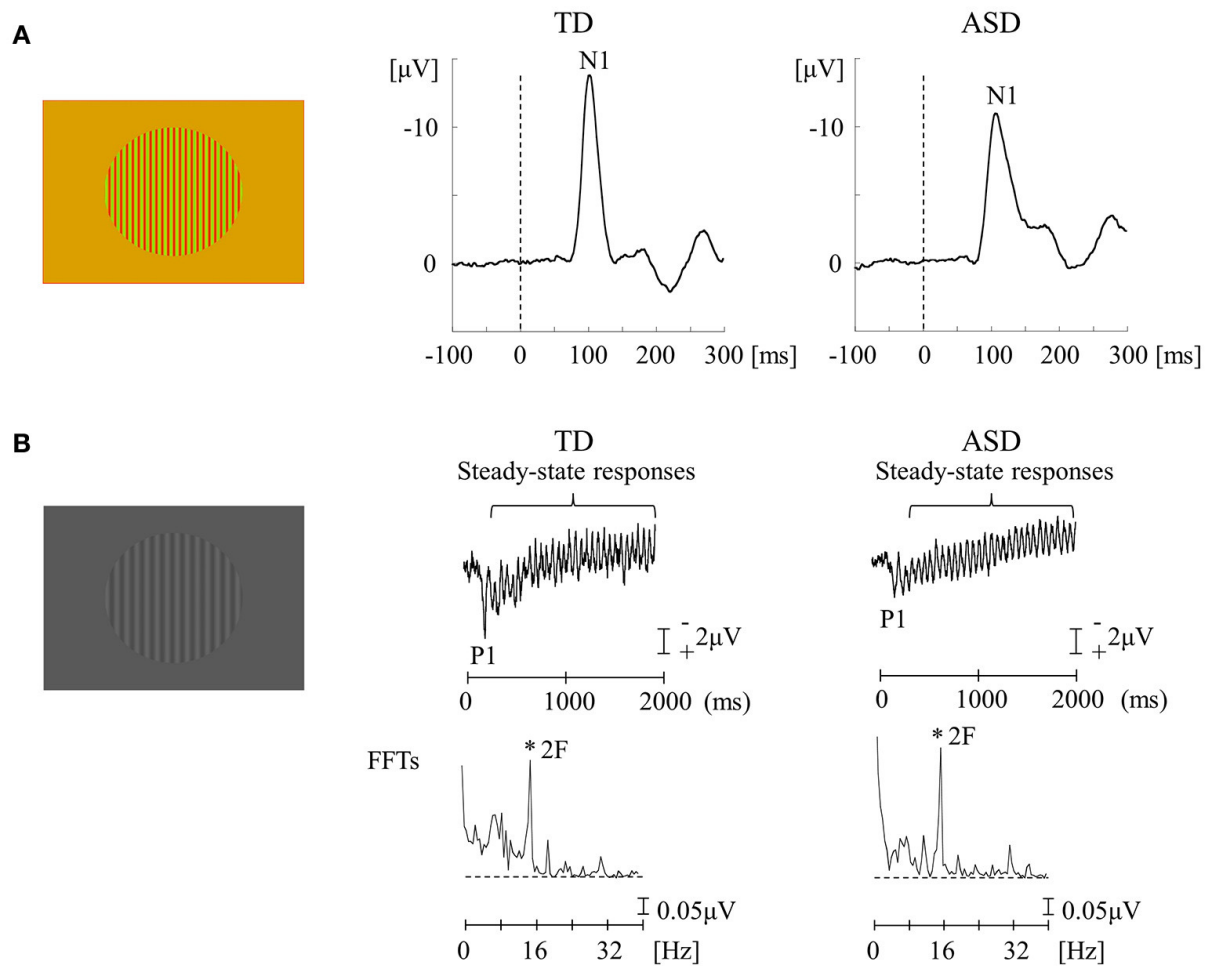
VEPs in response to RG and BW stimuli in the TD and ASD groups. **(A)** For RG stimuli (isochromatic, SF: 2.0 cpd), the mean N1 latency in the ASD group was significantly longer than that in the TD group. **(B)** For BW stimuli [low-contrast (16.6%), SF: 1.0 cpd, 8 Hz (16 reversals/s)], there was no obvious difference in the distribution of P1 and steady-state responses between the two groups. VEPs, visual evoked potentials; RG, red/green; BW, black/white; TD, typically developing; ASD, autism spectrum disorder; cpd, cycles per degree; SF, spatial frequency; FFTs, fast Fourier transforms; 2F, second harmonic (Modified from Fujita et al., 2011 with permission).

The functional characteristics of the P pathway are summarized as follows: high spatial frequency (SF) selectivity (P-interblob), color sensitivity (P-blob), low contrast sensitivity, poor temporal resolution, and slow conduction velocity (Livingstone and Hubel, 1988; Tobimatsu and Celesia, 2006; Table 1). Therefore, using red/green (RG) chromatic sinusoidal gratings with isoluminance of red and green are appropriate stimuli for the P pathway (P-blob [color] pathway; Figure 3A). This type of stimulus elicits the occipital N1 component (about 120 ms; Yamasaki and Tobimatsu, 2012; Yamasaki et al., 2014). The characteristics of the M pathway are summarized as follows: high contrast sensitivity, good temporal resolution, fast conduction velocity, color insensitivity, and low SF selectivity (Table 1). Accordingly, low contrast achromatic (black/white, BW) sinusoidal gratings with a frequency of 8 Hz (16 reversals/s) are appropriate stimuli (Figure 3B). This type of the stimulus evokes the occipital P1 component (about 120 ms), followed by steady-state responses (Yamasaki and Tobimatsu, 2012; Yamasaki et al., 2014).

The functions of lower-level P and M pathways at V1 were examined in adults with high-functioning ASD and typically developing (TD) adults (Fujita et al., 2011; Yamasaki et al., 2014). When testing the P pathway function, an unexpected finding was that the N1 latency for RG stimuli was more significantly delayed in ASD adults than in the TD controls (Figure 3A). RG stimuli could preferentially stimulate the P-blob but not the P-interblob pathway because the latter pathway responds better to stimuli with high SF and contrast (Table 1; Tobimatsu and Celesia, 2006). Thus, this finding suggests that the pathology of ASD implicates impairment of the P-blob pathway at a relatively low level (Fujita et al., 2011). Earlier psychophysical studies (Franklin et al., 2008, 2010) have tested color vision in ASD children and demonstrated altered color perception (color memory, color search, and chromatic discrimination) in ASD without color deficits determined by Ishihara color plates (Franklin et al., 2008, 2010; Fujita et al., 2011). Therefore, we proposed the likelihood of P-blob (color) dysfunction with compensatory P-interblob (form)-biased function (detailed form perception; Fujita et al., 2011). This interpretation was supported by adequate evidence of excellent fine form perception in ASD (Dakin and Frith, 2005). However, these studies did not assess the function of the P-interblob pathway.

Regarding the function of the M pathway, high-temporal frequency BW stimuli were used and both TD and ASD adults showed an occipital P1 with quasi-sinusoidal waveforms corresponding to the reversal frequency of BW. However, no significant difference was found in either P1 or steady-state responses between the two groups (Figure 3B; Fujita et al., 2011; Yamasaki et al., 2014). Accordingly, it appears that the function of lower-level M pathway is intact in ASD adults. Some psychological studies have reported abnormal function of the M pathway at the higher level (dorsal stream) in ASD adults, with preserved function of the M pathway at the lower level (Bertone et al., 2003; Pellicano et al., 2005), further supporting our VEP results (Fujita et al., 2011) that showed normal lower-level M pathway activity.

### Ventral stream function in ASD

Based on the unexpected finding of P-blob pathway impairment in ASD (section Parvocellular and Magnocellular Functions at V1 in ASD), we further investigated the methods by which the P-interblob and P-blob pathways within the ventral stream at the lower (V1) and higher (V4) levels are functionally abnormal in high-functioning adults with ASD (Yamasaki et al., 2017).

**Figure 4:**
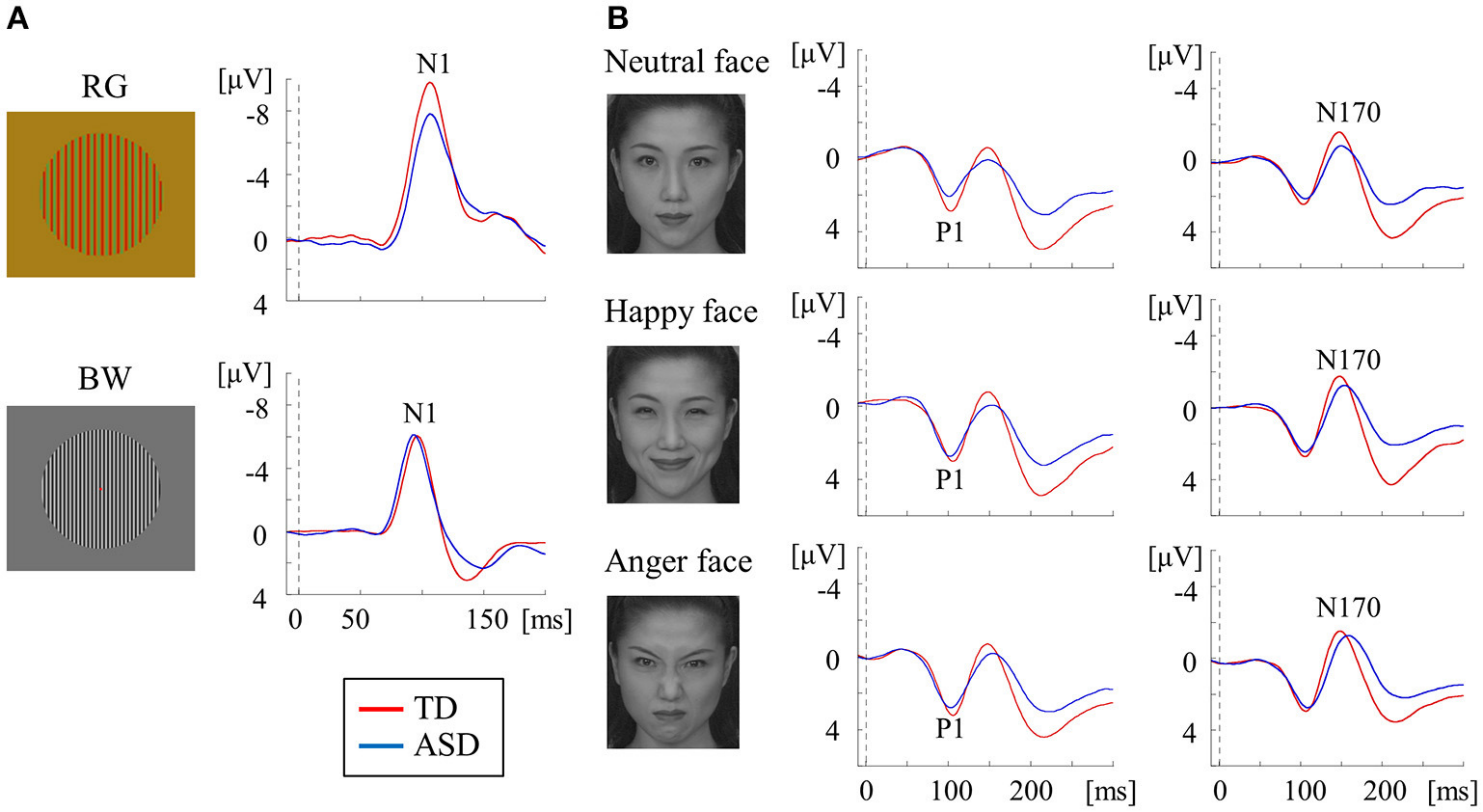
VEPs in response to RG, BW, and face stimuli in the TD and ASD groups. **(A)** For RG stimuli (isochromatic, SF: 2.0 cpd), compared with the TD group, the mean N1 latency was significantly prolonged in the ASD group. In contrast, for BW stimuli [high-contrast (98.8%), SF: 5.3 cpd], the mean N1 latency was significantly shorter in the ASD group than in the TD group. **(B)** For face stimuli (neutral, happy, and angry faces), the mean P1 latency in the ASD group was significantly shorter than that in the TD group, regardless of facial expression, while the mean N170 latency was significantly longer in the ASD group than in the TD group (Modified from (Yamasaki et al., 2017) with no permission was required to reproduce).

We used three types of visual stimuli (Figures 4A,B). An isoluminant chromatic RG pattern with medium SF (2.0 cycles/degree, cpd) and high-contrast achromatic BW gratings with high SF (5.3 cpd) could preferentially stimulate the P-blob and P-interblob pathways at the V1 level, respectively. The occipital N1 is a major component of responses to both stimuli in Figure 4A (Tobimatsu et al., 1996; Yamasaki et al., 2017). In addition, face stimuli are helpful to sequentially evaluate the form pathway from V1 to V4. The right-lateralized occipital P1 (V1 origin) and occipito-temporal N170 (V4 origin) are the major components of face VEPs (Figure 4B; Goto et al., 2005; Deffke et al., 2007; Nakashima et al., 2008; Mitsudo et al., 2011). P1 reflects the low-level features of the visual stimuli including the contrast, luminance, and SF (Rossion and Jacques, 2008). The N170 component reflects the processing of facial features or facial identification within the fusiform face area (Bentin et al., 1996).

In a study measuring RG VEPs, ASD adults exhibited prolonged N1 latency compared with TD adults. This indicates that the function of the P-blob pathway at V1 is impaired in adults with ASD (Figure 4A; Yamasaki et al., 2017). These results exhibited vigorous reproducibility even though ASD participants in this study were completely different from those in our earlier VEP study (Fujita et al., 2011; section Parvocellular and Magnocellular Functions at V1 in ASD). In BW VEPs, a significantly shorter N1 latency was found in adults with ASD adults than in TD adults (Figure 4A), suggesting enhanced P-interblob pathway function in V1 in ASD (Yamasaki et al., 2017). As the BW stimulus used in our study (Yamasaki et al., 2017) comprised high SF information (5.3 cpd), a shorter N1 latency was thought to reflect superior fine-form (high SF) processing at V1 in ASD. These findings further support our prediction of P-blob (color) dysfunction with P-interblob (form)-biased function in ASD (section Parvocellular and Magnocellular Functions at V1 in ASD).

For face VEPs, ASD adults showed a significantly faster P1 latency (V1 origin) than TD adults (Figure 4B), which suggested enhanced V1 function (Yamasaki et al., 2017). Neuropsychological studies have reported that ASD individuals may be more biased in favor of high SF than low SF in face recognition (Deruelle et al., 2004, 2008). Moreover, a previous VEP study demonstrated that the emotional effect on P1 was significant for high SF, but not low SF, in an ASD group (Vlamings et al., 2010). As the face stimuli used in our study (Yamasaki et al., 2017) were composed of broadband SF, superior V1 function could reflect better fine-form perception related to the local high SF processing of faces in V1 in ASD adults. This finding is concordant with the results of study using BW VEPs (Figure 4A; Yamasaki et al., 2017).

Adults with ASD were also found to exhibit prolonged N170 latency in response to face stimuli (Figure 4B). In addition, a greater difference in latency between P1 and N170 and between N1 for BW and N170 (i.e., prolonged cortico-cortical conduction time between V1 and V4) was found in ASD adults. Therefore, we concluded that individuals with ASD exhibit impaired global face processing owing to deficits in integrating multiple local high SF facial information processing at V4 (Yamasaki et al., 2017).

Overall, adults with ASD exhibit enhanced P-interblob (form; local high SF) processing but impaired P-blob (color) processing in V1. Moreover, they show poor gestalt face processing due to insufficiencies in incorporating multiple sources of local high SF facial information at V4 (Yamasaki et al., 2017).

### Dorsal stream function in ASD

**Figure 5:**
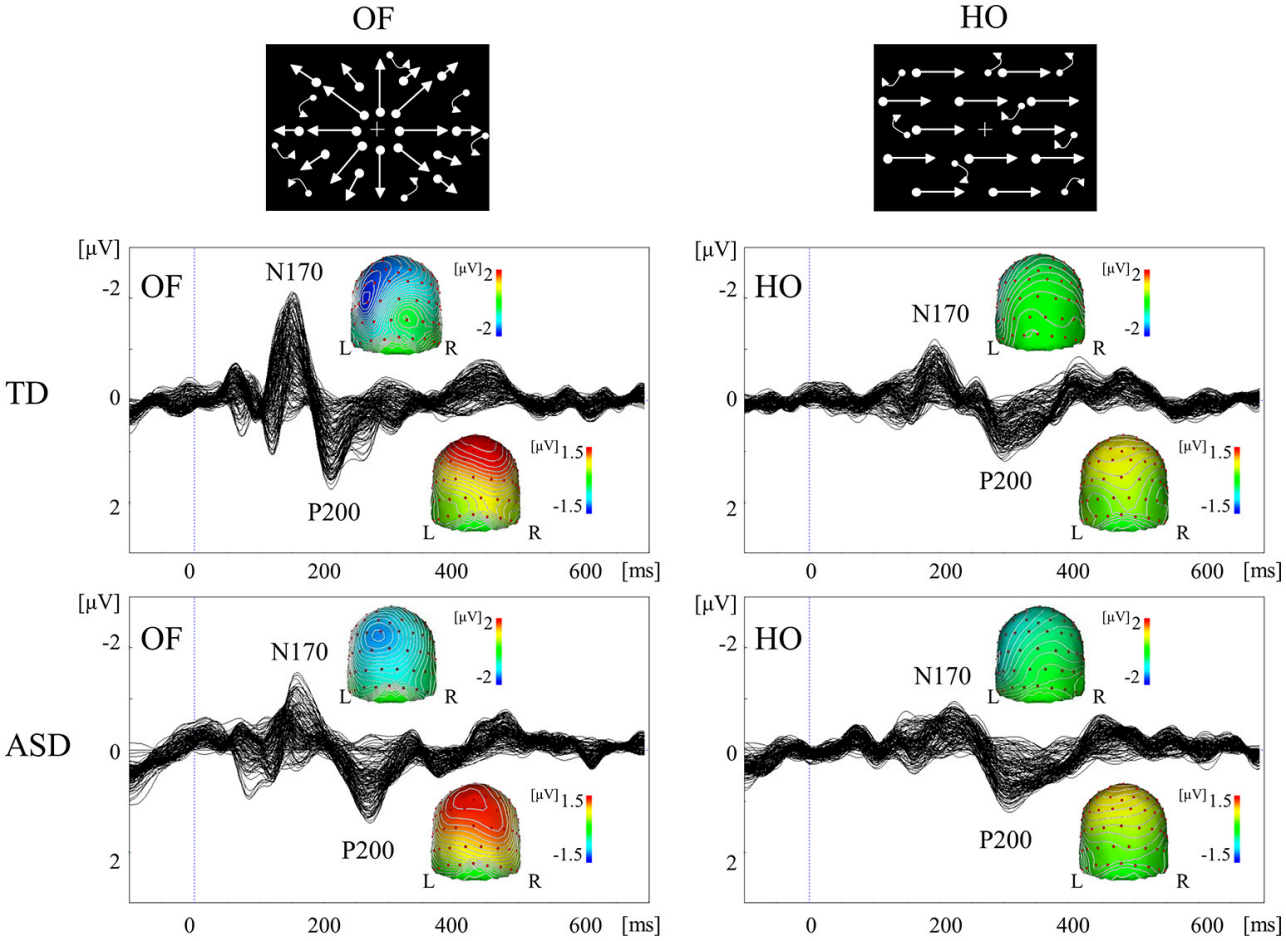
VEPs in response to coherent OF and HO motion in the TD and ASD groups. The ASD group displayed significantly prolonged N170 and P200 latencies in response to OF, but not HO, stimuli, compared with the TD group. OF, optic flow; HO, horizontal motion (Modified from Yamasaki et al., 2011b with permission).

Coherent motion stimuli using random dots are useful in the investigation of higher-level dorsal function (Yamasaki and Tobimatsu, 2012; Yamasaki et al., 2014). Radial optic flow (OF) and horizontal motion (HO) are the most commonly used in exploring global motion (Figure 5). The occipito-temporal N170 (V5/MT origin) and parietal P200 (parietal lobe origin) are evoked by these stimuli in VEPs. We found that OF is largely handled by the v-d stream including IPL, whereas HO is mainly processed in the d-d stream including SPL in healthy subjects using functional magnetic resonance imaging (fMRI; Yamasaki et al., 2011a, 2012a,b).

A comparison of coherent OF and HO perception in adults with ASD and TD (Yamasaki et al., 2011b, 2014) showed comparable VEP responses and scalp maps between the two groups. Conversely, ASD exhibited the significant delayed latencies of N170 and P200 components in response to OF, but not HO, stimuli (Figure 5). These findings indicated the selective impairment of OF perception, which was associated with higher-level dorsal (the v-d pathway) function in ASD adults despite the fact that psychophysical thresholds were preserved (Yamasaki et al., 2014). OF is a type of complex motion characterized by multidirectional movement with depth, which is crucial for the navigation of self-movement (Gibson, 1950). OF information is also basis of action-related information including biological motion and facial expressions. Thus, OF processing is related to the dorsal stream (the v-d stream) function that is necessary to perceive the outer world, including other individuals. Consequently, the specific dysfunction of this pathway could underlie the social impairment observed in ASD. Furthermore, children with ASD demonstrated postural hyporeactivity to visually perceived environmental motion (visuo-postural tuning) compared with children with TD (Gepner and Mestre, 2002), which partially supports the notion that OF perception is specifically reduced in ASD individuals (Yamasaki et al., 2011b, 2014).

### Subliminal face processing at V1 in ASD

Conscious perception of (supra-threshold) face evokes the occipital N1 (about 100 ms), P1 (about 120 ms), and the occipito-temporal N170 (about 170 ms). N1 and P1 components are originated in V1 while N170 generated from V4 (Bötzel et al., 1995; George et al., 1996; Fujita et al., 2013). If a supra-threshold face is presented upside down (the so-called “face inversion effect”), N170 reveals augmented amplitude and prolonged latency (Jacques et al., 2007). This phenomenon reflects the disrupted integration of features into a gestalt whole or holistic face representation (Young et al., 1987). We have previously described that in healthy subjects, the amplitudes of occipital P1 for unrecognizable (subliminal) faces are significantly greater than those for objects in the upright position (Mitsudo et al., 2011). In contrast, a significant reduction was found in P1 amplitudes for inverted faces compared with those of upright faces. This effect is quite opposite to the face inversion effect for supra-threshold stimuli and is called the “subliminal face effect (SFE)” (Fujita et al., 2013). Therefore, it is likely that faces and objects are processed in a different way at the V1 level, even when subjects are unaware of stimuli before face-specific processing occurs within the fusiform gyrus (V4). Overall, alterations of N1 or P1 in response to subliminal upright and inverted faces can offer insight into the neural mechanism of automatic face processing in high-functioning individuals with ASD (Fujita et al., 2013).

**Figure 6:**
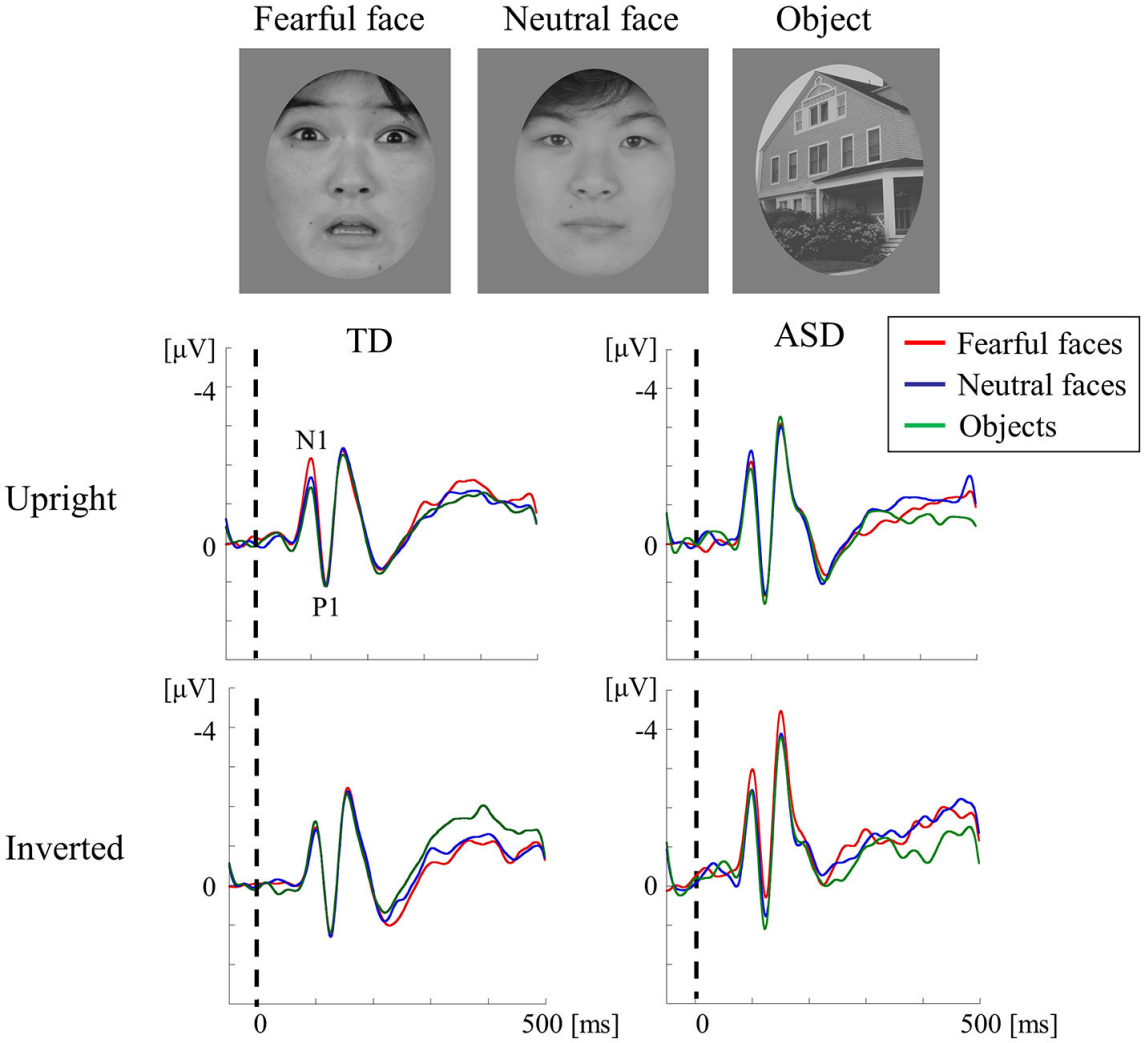
VEPs in response to subliminal face stimuli using a backward-masking paradigm in the TD and ASD groups. Faces (neutral and fearful faces) and objects were randomly presented for 20 ms (sub-threshold duration) in an upright or inverted orientation, followed by a 1,000-ms pattern mask. The target appeared in 10% of the trials in each block and was presented for 600 ms to draw the participant's attention away from the experimental stimuli. In both groups, the stimuli (in both orientations) elicited a negative component at ~100 ms (N1) and a following positive peak at ~120 ms (P1) after stimulus onset. The TD group exhibited a fearful-specific SFE in the upright condition. In contrast, adults with ASD exhibited no signs of SFE, as reflected by the unaltered N1 for stimuli of different types and orientations. SFE, subliminal face effect (Modified from Fujita et al., 2013 with permission).

We recorded VEPs in response to visual stimuli with upright and inverted faces (fearful and neutral) and objects that were presented subliminally to ASD and TD adults using a backward-masking paradigm (Fujita et al., 2013; Figure 6). The TD adults exhibited a fearful-specific SFE in the upright condition. Conversely, adults with ASD displayed a lack of SFE, as shown by the unchanged N1 for stimuli of different types and orientations (Figure 6). The TD adults did not show upright SFE for neutral faces, which implies that at least emotional face information is altered in ASD adults. Therefore, ASD appear to exhibit different automatic visual processing for emotional faces in V1 level (Fujita et al., 2013). In TD adults, a fearful face-specific SFE can be based on the effect of low SF information on faces (Mitsudo et al., 2011), which is processed by the M pathway (Tobimatsu and Celesia, 2006). However, in our previous VEP study (section Parvocellular and Magnocellular Functions at V1 in ASD), the lower-level M pathway function was maintained in ASD adults during the perception of high-temporal frequency BW stimuli (Fujita et al., 2011). Thus, a lack of fearful face-specific SFE in ASD may reflect the impairment of the M pathway from layers 4Cα and 4B to the P-blob region within V1, but not from layer 4Cα to the thick stripes of V2 and V5/MT.

## Visual attention in ASD

### Concept of attention networks

Selective attention is the cognitive process of focusing on a particular aspect of information. There exists two major mechanisms: bottom-up attention and top-down attention. Bottom-up attention is automatically produced or driven by the properties of stimuli. In contrast, top-down attention denotes the volitional attention, which focuses on a location and/or an object on the basis of current behavioral goals (Ciaramelli et al., 2008). These attention mechanisms can drive in parallel, although bottom-up attention arises more quickly than top-down attention (Treisman et al., 1992; Maekawa et al., 2011).

The following two attention networks have been described in the human brain: ventral attention network (VAN) and dorsal attention network (DAN) (Corbetta et al., 2008; Vuilleumier, 2013; Farrant and Uddin, 2016; Figure 2B). The VAN contains key nodes in the temporoparietal junction and ventral frontal cortices, which are related to bottom-up attention. In contrast, the DAN consists of key nodes in the bilateral intraparietal sulcus, SPL, and frontal eye fields, which are associated with top-down attention (Farrant and Uddin, 2016). The cortical projections of three branches of SLF overlap with VAN and DAN nodes (Figure 2A). The SLF I connects brain regions within the DAN. The SLF II connects the VAN's parietal regions of the VAN with the DAN's prefrontal regions, allowing these two networks to communicate. The SLF III connects regions within the VAN. The SLF III is larger on the right than the left, while the SLF I is symmetrical; the SLF II tends to be larger in the right hemisphere (Lunven and Bartolomeo, 2017).

### ERP study: bottom-up and top-down attention in ASD

The ERP helps capture neural activity related to both sensory and cognitive process. Two specific ERP components, the visual mismatch negativity (MMN) and visual P300 (or P3), are candidate biomarkers for bottom-up and top-down attention, respectively (Maekawa et al., 2011; Yamasaki et al., 2014). The visual MMN component reflects the pre-attentive, automatic processing of visual stimuli. It is obtained by subtracting waveforms elicited by frequently occurring, standard stimuli from infrequently occurring, deviant stimuli presented in an oddball paradigm. The visual MMN is defined as negativity measured at occipital electrodes between 150 and 350 ms after the onset of a deviant visual stimulus. The neural origin of the visual MMN has been considered to originate from the prestriate and prefrontal areas (Maekawa et al., 2012). The P300 response reflects higher-level attention and memory-related processing. It arises ~300 ms after presenting a stimulus and is elicited when discriminating deviant and standard stimuli. The P300 response has two subcomponents that are related to either passive or active attention. The P3a component reflects the involuntary attention switching to stimuli. In contrast, the P3b component underlies the active response (active attention) to stimuli (Samson et al., 2006; Yamasaki et al., 2014).

**Figure 7:**
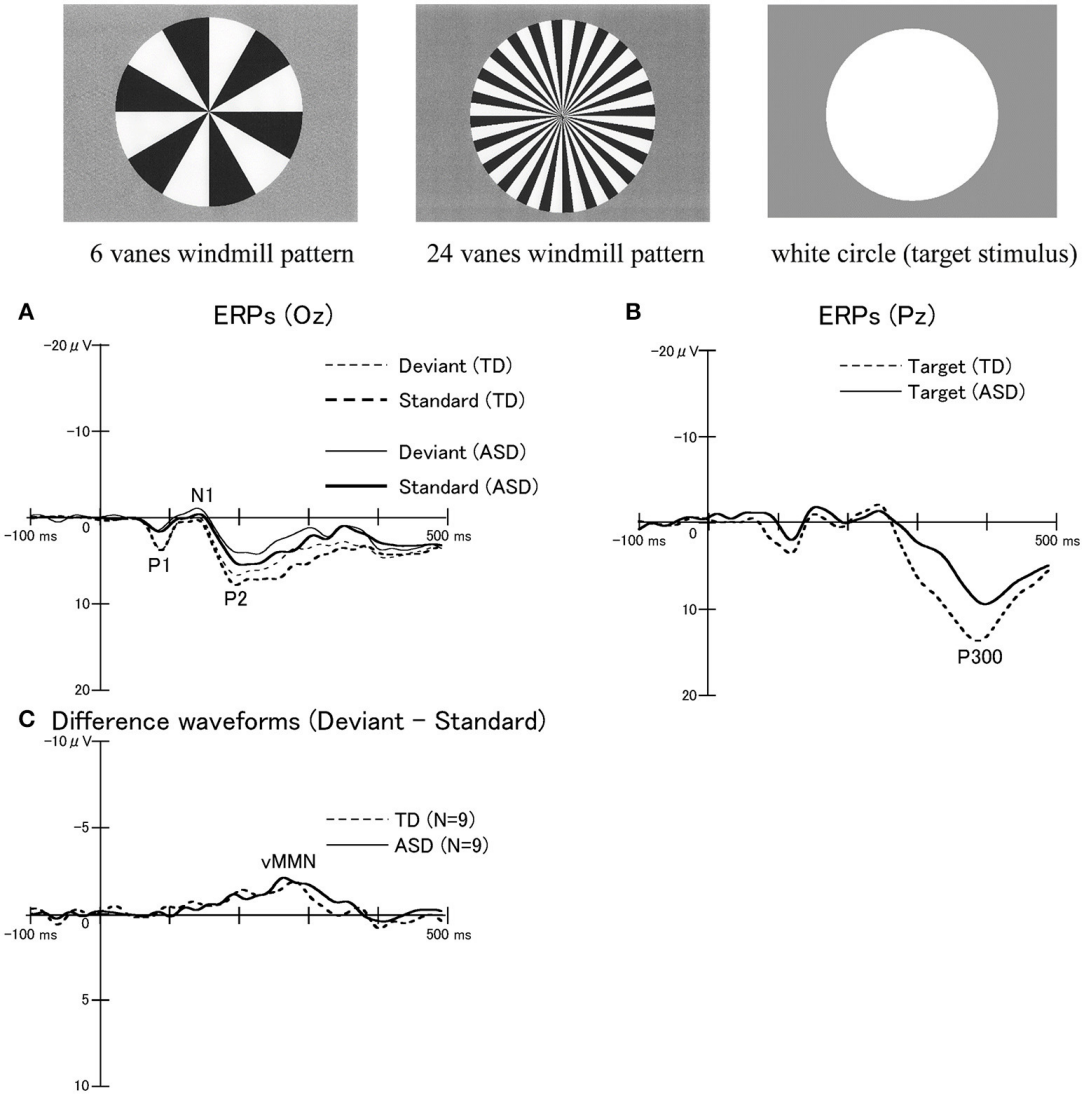
ERPs in response to windmill pattern stimuli using an oddball paradigm in the TD and ASD groups. Three stimulus types were used to evoke ERPs using an oddball paradigm: circular black-white windmill pattern stimuli with 6 vanes and with 24 vanes and an unpatterned white circle stimulus. The two windmill pattern stimuli were adopted as standard or deviant stimuli (their probabilities changed between sessions). A white circle was always used as the target stimulus. Probabilities of standard, deviant, and target stimuli are 8:1:1, respectively. **(A)** Waveforms for standard stimuli (TD, thick dotted line; ASD, thick solid line) and for deviant stimuli (TD, thin dot line; ASD, thin solid line) at Oz. **(B)** Waveforms for target stimuli at Pz (TD, dotted line; ASD, solid line). While there were no significant differences in P300 latencies between the two groups, the P300 amplitudes in the ASD group were significantly smaller than those in the TD group. **(C)** Differences in waveforms from responses to standard stimuli relative to responses to deviant stimuli at Oz (TD, dotted line; ASD, solid line). There were no statistically significant differences in the mean peak latency and amplitude of visual MMN between the two groups. MMN, mismatch negativity (Modified from Yamasaki et al., 2014 with permission).

We performed ERPs to examine the function of bottom-up (VAN) and top-down (DAN) visual attention systems in ASD and TD adults (Maekawa et al., 2011; Yamasaki et al., 2014). The oddball paradigm using windmill pattern stimuli (Figure 7) was applied to ERPs, which produced MMN (bottom-up attention), P300 (top-down attention), and P1 (lower level processing) components. Interestingly, ASD adults detected the target much faster than TD controls. Nevertheless, there was no significant difference in the MMN response between the two groups (Figure 7C). On the contrary, the reduction of the P1 and P300 amplitudes with the prolonged P300 latency were observed in ASD adults compared to TD adults (Figures 7A,B). Hence we concluded that intact bottom-up attention (MMN), which was related to the VAN, may contribute to the greater performance of simple task in ASD individuals though lower-level processing (P1) and top-down attention (P300) were impaired (Maekawa et al., 2011, 2012; Yamasaki et al., 2014).

## DTI studies in ASD

DTI provides a measure of structural connectivity by measuring the diffusion of water molecules in the brain to reconstruct white matter tracts (Hernandez et al., 2015). The DTI indices that are commonly reported include the following: (1) fractional anisotropy (FA) quantifies the degree of the directionality of water diffusion, ranging 0 (random) to 1 (unidirectional); (2) mean diffusivity (MD) represents average amount of water diffusion within a given voxel; and (3) axial (parallel), and (4) radial (perpendicular) diffusivity are indices of water molecule movement running parallel and perpendicular to the principle axis of diffusion, respectively (Ameis and Catani, 2015). These DTI indices provide us information about the integrity and architectural organization of the underlying white matter microstructure (Ameis and Catani, 2015). Lower FA and higher MD values usually imply damaged or impaired fiber integrity, which is attributable to increased diffusion and loss of coherence in the preferred movement direction (Soares et al., 2013). Reduced axial diffusivity values suggest a decline in axonal integrity, and decreased radial diffusivity values may provide a non-invasive surrogate marker of demyelination. These values reflect subtle structural abnormalities that cannot be detected by FA (Papadakis et al., 1999; Noriuchi et al., 2010).

We conducted DTI analysis in high-functioning children with ASD and TD controls (Noriuchi et al., 2010). DTI revealed the significant reduction of FA and axial diffusivity values in the several regions of white matter in the ASD subjects compared to TD controls. These regions included the white matter around the left dorsolateral prefrontal cortex (DLPFC), temporoparietal junction, right temporal pole, amygdala, SLF, and IFOF, which were related to the parallel visual pathways and attentional networks. In the left DLPFC, a negative correlation was found between the FA value in this area and the degree of social impairment in children with ASD. The cerebellar vermis lobules showed higher axial diffusivity values in the ASD group. These DTI findings and their relationship with social impairment provide additional evidence of functional abnormalities in cerebral and cerebellar white matter in ASD (Noriuchi et al., 2010).

Our DTI findings are in accordance with numerous DTI studies from other research groups in children, adolescents, and adults with ASD (Travers et al., 2012; Ameis and Catani, 2015; Hernandez et al., 2015; Rane et al., 2015; Ismail et al., 2016; Li et al., 2017). These studies have documented multiple structural connectivity differences, mostly suggesting reduced white matter integrity (lower FA and/or higher MD values) in long-range anterior-posterior and interhemispheric fiber tracts. Notably, many specific fiber tracts reported to be altered in ASD serve as structural connections among the brain areas related to social cognition (Hernandez et al., 2015). The most commonly reported areas for decreased FA values are association fibers (the most common being the SLF, IFOF, uncinate fasciculus, ILF, and cingulum) and the corpus callosum. The SLF, corpus callosum, and corticospinal tract are the most commonly mentioned structures in reports of increased MD in ASD (Rane et al., 2015).

Few studies have directly studied the relationship between DTI and visual perception in ASD. One DTI study examined white matter pathways involved in face processing in adolescents and adults with ASD and TD controls (Conturo et al., 2008). Radial diffusivity values were increased in amygdala-fusiform connections bilaterally and hippocampus-fusiform connections in the left side in individuals with ASD. In contrast, hippocampus-fusiform connections in the right side showed reduced radial diffusivity in ASD, which correlated with lower face recognition scores. These findings suggested an early functionally significant pathological process in the right hippocampus-fusiform pathway in accordance with small-diameter axons (with corresponding slower neural transmission) and/or higher packing density (Conturo et al., 2008). Another DTI study has demonstrated the significant alterations in the microstructural organization of white matter in the right IFOF, which is associated with an inferior visuospatial processing performance in ASD. The authors concluded that structural brain abnormalities may influence atypical visuospatial processing in ASD (McGrath et al., 2013a,b). Accordingly, it is likely that white matter connectivity (in particular, SLF, IFOF, and ILF) that are associated with parallel visual pathways and visual attention networks are often disrupted in ASD.

## Altered connectome functions in ASD

**Figure 8:**
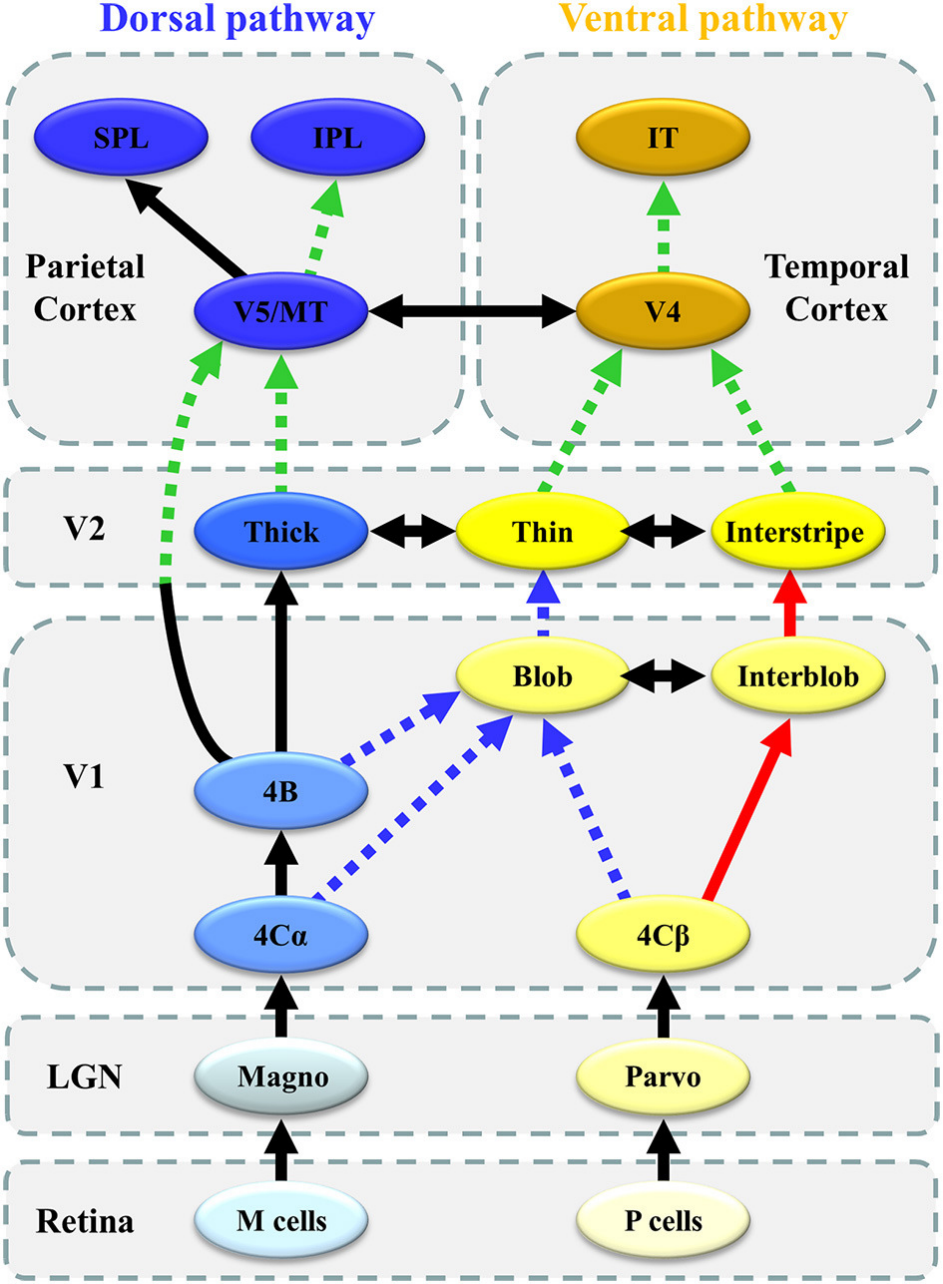
Schematic representation of altered connectivity of visual networks in ASD. Red thick solid arrows indicate enhanced processing (or overconnectivity), whereas blue broken arrows suggest impaired processing (or underconnectivity) in the local circuitry within V1. Green broken arrows indicate impaired global integration of local information (or long-range underconnectivity) between lower-and higher-level visual areas.

In our series of VEP and ERP studies, we found that the following: (1) enhanced and impaired processing co-exists within the lower visual area (V1) (sections Parvocellular and Magnocellular Functions at V1 in ASD and Ventral Stream Function in ASD), (2) local information integration from lower visual areas (V1) is impaired in higher-level visual areas after V4 and V5/MT (sections Ventral Stream Function in ASD and Dorsal Stream Function in ASD), and (3) the DAN is impaired while the VAN is intact in ASD (section ERP Study: Bottom-Up and Top-Down Attention in ASD; Figure 8). These complex functional alterations in visual and attention networks support the possibility of altered connectivity within and between distributed brain regions, instead of local deficits in specific brain areas in ASD (Müller, 2008).

The most important finding in our VEP studies was the co-existence of enhanced P-interblob and impaired P-blob pathway processing with an intact M pathway in V1 in individuals with ASD. Many behavioral studies have reported a preference for local processing in ASD (Dakin and Frith, 2005). An fMRI study demonstrated atypical increment of local functional connectivity in ASD in posterior brain areas including V1 (Keown et al., 2013). Thus, our VEP finding of an enhanced P-interblob pathway in ASD partly supports these previous behavioral and neuroimaging findings. Conversely, to date, no electrophysiological or neuroimaging studies have reported impaired P-blob function in V1 in ASD. Recent data have suggested that the alteration of connectivity within V1 is more complex than earlier suggestions of enhanced simple (local) processing in V1, thereby providing new insight into the neural circuit mechanisms underlying ASD.

At present, mechanisms underlying altered V1 connectivity in ASD remain unclear. We speculate that blob impairment is a key component of this mechanism. In V1, the P-blob stream is characterized by a higher mitochondrial CO concentration, whereas the P-interblob stream has a lower concentration of CO. CO is well-known to be the last enzyme in the respiratory chain of mitochondria that is related to oxidative metabolism and energy production (Liu and Wong-Riley, 1995; Bókkon and Vimal, 2010, 2013) and is closely coupled to neuronal functional activity (Liu and Wong-Riley, 1995). It is interesting to note that recent neuroimaging and postmortem studies have reported mitochondrial dysfunction in the brains of ASD individuals (Rossignol and Frye, 2012, 2014). Thus, deficits in the P-blob pathway in ASD may reflect mitochondrial dysfunction in the blob region of V1 (Yamasaki et al., 2017). This impairment in turn induces the compensatory enhancement of P-interblob function in ASD because the P-blob pathway anatomically interacts with the P-interblob pathway (Yabuta and Callaway, 1998). Further studies are needed to confirm this hypothesis using neuroimaging techniques that can separately evaluate the blob and interblob regions.

In contrast, behavioral studies have proposed that neuro-integrative processing at higher cortical levels in the ventral and dorsal pathways is impaired, while lower-level processing is spared (Bertone et al., 2003, 2005; Bertone and Faubert, 2006). Numerous fMRI studies have demonstrated that individuals with ASD had long-range underconnectivity with local overconnectivity (Courchesne and Pierce, 2005; Anderson et al., 2011; Keown et al., 2013). Many DTI studies, including ours (section DTI Studies in ASD), detected white matter alterations of the ventral (ILF and IFOF) and dorsal (SLF) streams in ASD (Rane et al., 2015). Therefore, our VEP findings of altered connectivity in parallel visual pathways between V1 and V4 or between V1 and V5/MT are consistent with the alterations of anatomical and functional connectivity of the ventral and dorsal streams revealed by DTI and fMRI studies in ASD.

Interestingly, our VEP studies using coherent motion revealed dissociative impairment within the dorsal stream along the SLF; the v-d stream (OF processing) was impaired with preserved d-d stream (HO processing) function in ASD (section Dorsal Stream Function in ASD). Furthermore, in our ERP study, the DAN (top-down) related to SLF I was impaired with an intact VAN (bottom-up) related to SLF III in ASD (section ERP Study: Bottom-Up and Top-Down Attention in ASD). Thus, connectivity impairment may be anatomically uneven within the SLF. Alternatively, SLF connectivity impairment may be exhibited in situations requiring more complex processing (OF processing vs. HO processing; top-down vs. bottom-up).

As mentioned above, this review suggests that altered functional connectivity within visual and attention networks exists in ASD. However, it remains unknown (1) how the strength and direction of functional connectivity within these networks differ between ASD and TD, and (2) how the altered networks influence the social cognition network in ASD. In the field of network neuroscience, a mathematical modeling approach (graph-theoretical approach) is used for whole-brain functional connectivity analysis on the basis of resting-state fMRI and magnetoencephalography data (Uehara et al., 2014; Bassett and Sporns, 2017). This approach can reveal the strength and direction of whole-brain functional connectivity in detail. From our VEP and ERP data, the different visual stimuli may induce differential effects on whole-brain functional network in ASD. Therefore, further studies using network neuroscience methods on functional connectomes in the ASD brain under visual task-related condition will be needed to understand the neural mechanisms of atypical visual perception and social impairment in ASD.

## Conclusion

Recent data have revealed that unusual visual perception observed in ASD may result from alterations to the local circuitry within the visual area and connectivity between distributed visual cortical areas as well as the connectivity of attention networks. Therefore, we conclude that the altered connectivity of visual processing networks may contribute to impaired social communication exhibited in ASD. The underlying pathophysiological mechanism of ASD can be considered a “connectopathy.”

## Author contributions

Conceived and designed the experiments: TY, TM, TF, and ST. Performed the experiments: TY, TM, and TF. Analyzed the data: TY, TM, and TF. Wrote the paper: TY, TM, TF, and ST.

### Conflict of interest statement

The authors declare that the research was conducted in the absence of any commercial or financial relationships that could be construed as a potential conflict of interest.
